# Stomatal Responses of Two Drought-Tolerant Barley Varieties with Different ROS Regulation Strategies under Drought Conditions

**DOI:** 10.3390/antiox12040790

**Published:** 2023-03-23

**Authors:** Xiachen Lv, Yihong Li, Rongjia Chen, Mengmeng Rui, Yizhou Wang

**Affiliations:** 1 Institute of Crop Science, College of Agriculture and Biotechnology, Zijingang Campus, Zhejiang University, Hangzhou 310058, China; 2 Zhejiang Provincial Key Laboratory of Crop Germplasm, Zhejiang University, Hangzhou 310058, China

**Keywords:** barley, guard cell, ROS, regulation strategies, stomata, water use efficiency

## Abstract

Drought stress is a major obstacle to agricultural production. Stomata are central to efforts to improve photosynthesis and water use. They are targets for manipulation to improve both processes and the balance between them. An in-depth understanding of stomatal behavior and kinetics is important for improving photosynthesis and the WUE of crops. In this study, a drought stress pot experiment was performed, and a transcriptome analysis of the leaves of three contrasting, cultivated barley genotypes Lumley (Lum, drought-tolerant), Golden Promise (GP, drought-sensitive), and Tadmor (Tad, drought-tolerant), generated by high-throughput sequencing, were compared. Lum exhibited a different WUE at the leaf and whole-plant levels and had greater CO_2_ assimilation, with a higher *g_s_* under drought stress. Interestingly, Lum showed a slower stomatal closure in response to a light–dark transition and significant differences compared to Tad in stomatal response to the exogenous application of ABA, H_2_O_2_, and CaCl_2_. A transcriptome analysis revealed that 24 ROS-related genes were indeed involved in drought response regulation, and impaired ABA-induced ROS accumulation in Lum was identified using ROS and antioxidant capacity measurements. We conclude that different stomatal ROS responses affect stomatal closure in barley, demonstrating different drought regulation strategies. These results provide valuable insight into the physiological and molecular basis of stomatal behavior and drought tolerance in barley.

## 1. Introduction

Over the last decade, global losses in crop production due to drought are estimated to have been approximately USD 30 billion [1]. Frequent and intensive drought has become one of the crucial factors restricting agricultural production in the context of climate change, posing a serious challenge to global food security [2]. Therefore, understanding the water use characteristics of major *Poaceae* crops, which provide more than 60% of the world’s food production, is essential for promoting agricultural water resource utilization and for the improvement of crop water use efficiency (WUE) in response to drought conditions [3]. Among cereals, barley (*Hordeum vulgare*) is an important grain crop that ranks fourth after maize (*Zea mays*), rice (*Oryza sativa*), and wheat (*Triticum aestivum*) in terms of total production around the world [4]. It is susceptible to drought during the growing season, which affects its relative water content (RWC), photosynthetic rate (*A*), stomatal conductance (*g_s_*), transpiration rate (*E*), leaf temperature (*T_leaf_*), and other physiological traits [5]. Drought also leads barley to produce reactive oxygen species (ROS) that cause oxidative damage, resulting in membrane lipid peroxidation and seriously affecting normal physiological activities which, in turn, causes a yield reduction [6,7,8,9,10].

In response to drought, plants counteract a water deficit through physiological processes such as photosynthesis, osmoregulation, and ROS scavenging [11,12]. Under drought stress, one of key response mechanisms of plants is the induction of the endogenous hormone abscisic acid (ABA), which acts as a major regulator and activates a series of cellular signaling pathways that lead to the rapid closure of stomata to prevent water loss and cell dehydration [13,14,15,16]. The signaling pathway for ABA-induced stomatal closure includes the non-calcium-dependent protein kinase OPEN STOMATA 1 (OST1) and the calcium-dependent signaling cascades. In the former, ABA receptors (PYR/PYL/RCAR) bind to ABA and collectively inhibit the activity of 2C-type protein phosphatases (PP2Cs), releasing the protein kinase OST1 and regulating potassium (K^+^) and anion (Cl^−^) channel activity [15,17]. In the latter, ABA modulates the cytosolic free calcium concentrations ([Ca^2+^]_i_) by regulating the Ca^2+^ influx from the plasma membrane, and the production of ROS, mediated by NADP(H) oxidase, is activated [18,19,20,21,22]. This affects K^+^ and Cl^−^ channel activity, thereby decreasing guard cell turgor pressure and leading to stomatal closure [22].

Stomatal behavior has a major impact on global carbon and hydrological cycles. In addition to enabling CO_2_ influx into plant leaves for photosynthesis, stomata also play a dual and often conflicting function in limiting water loss through transpiration [23,24]. Not surprisingly, stomatal conductance is tightly correlated with WUE in plants. WUE is defined as the amount of dry matter accumulated per unit of water consumed by transpiration in plants. At the leaf scale, WUE can also be calculated as the ratio between *A* and *E*. Early studies have shown that WUE and *A* are directly related to stomatal function [25,26,27,28]. The coordination between the photosynthetic carbon gain and stomatal behavior is key to determining the WUE; however, mutations that increase the WUE often come at the cost of carbon assimilation, reflecting the trade-off between CO_2_ availability and reduced stomatal water loss. For example, in *Arabidopsis* mutants, a reduced *g_s_* with a loss of function in the vesicle-trafficking protein SYP121 resulted in a greater water utilization but a reduced CO_2_ assimilation, affecting plant growth [27]. Similarly, Antunes et al., 2012, showed that a decrease in sucrose synthase 3 (SuSy3) in *Solanum tuberosum* resulted in an increase in the WUE, while a reduction in *g_s_* restricted the rate of CO_2_ assimilation [29]. In addition, Wang et al., 2014, found enhanced, light-induced stomatal opening rates and improved growth rates in overexpressed H^+^-ATPase plants [30]. However, such changes have led to a large stomatal aperture which reduces the WUE. In fact, alterations to the *g_s_* that increase photosynthesis can do so at the expense of water loss. Intriguingly, the stomatal response to environmental changes is much slower than *A* [31,32,33,34]. This nonsynchronous response between a rapid photosynthetic rate and sluggish stomatal movement results in plants having a suboptimal intrinsic WUE (iWUE = *A*/*g_s_*) [35]. To enhance the iWUE, accelerating the rate of stomatal response can be regarded as a potential choice. Recently, researchers expressed a synthetic K^+^ channel, BLINK1, in guard cells that accelerated the kinetics of stomatal opening and closing, providing a promising way to improve WUE without the penalty of carbon fixation [36]. It can be predicted that plants with more rapid stomatal kinetics facilitate a greater CO_2_ uptake and avoid unnecessary water loss and therefore have a higher iWUE [37]. Therefore, an in-depth understanding of stomatal behavior, aperture regulation, and kinetics is of great significance to improving the photosynthesis and WUE of crops.

ROS (e.g., O_2_^−^, H_2_O_2_, OH^−^, and ^1^O_2_) function as signaling molecules in cells, but are also considered to be unavoidable, toxic byproducts of aerobic metabolism [38,39]. Intracellular superoxide (O_2_^−^) is mainly generated through the oxidation of NADPH by NADPH oxidase enzymes (RBOH/NOXs) or by electron leakage from mitochondrial aerobic respiration. Superoxides are rapidly converted into hydrogen peroxide (H_2_O_2_) by specific superoxide dismutases (SOD). H_2_O_2_ is able to oxidize cysteine residues on proteins to initiate redox biology. In addition, H_2_O_2_ may be converted to H_2_O by cellular antioxidant proteins such as peroxiredoxins (PRX), glutathione peroxidase (GPX), and catalase (CAT). When H_2_O_2_ levels rise uncontrollably, hydroxyl radicals (OH^−^) are formed through reactions with metal cations (Fe^2+^) and irreversibly damage cellular macromolecules [40]. By controlling the toxicity of ROS, plants have been able to use ROS as signaling molecules to coordinate a wide range of environmental and developmental processes. For instance, ROS play an important role in growth and development by triggering cell division and cell death. They are also involved in signaling during responses to biotic and abiotic factors [41,42]. Since many enzymatic components of the plant antioxidant system are well characterized, perturbing this scavenging system is a great strategy for investigating ROS signaling [43]. For example, peroxisomal catalases are major scavengers of H_2_O_2_, and a reduction in their levels allows for the H_2_O_2_ concentration in plants to be modulated. Under conditions that promote photorespiration, such as high light, the subcellular production of ROS is substantially elevated, and ROS are readily scavenged by various antioxidant systems. Conversely, catalase-deficient plants accumulate photorespiratory H_2_O_2_, leading to an elaborate transcriptional response [44,45,46]. ROS are involved in the regulation of abscisic acid (ABA)- [15,22,47], ethylene- [48], methyl jasmonate- [20] and salicylic-acid-mediated stomatal signaling [49]. The rapid induction of ROS during CO_2_-induced stomatal closure has also been found in the abaxial epidermis of *Arabidopsis* [50]. A key player in this network of ROS-producing enzymes is the specialized respiratory burst, or RBOH [51]. The NADPH oxidase mutant *AtrbohD*/*AtrbohF* impairs ROS production and calcium channel (I_Ca_) activation in response to ABA [19], which reveals the tight relationship between Rboh-dependent ROS and calcium homeostasis. Collectively, ROS are central intermediate signaling components in plant guard cells.

Lumley (Lum), a cultivated barley genotype that is originally from Nepal, exhibits a high drought tolerance. However, its drought tolerance mechanism is still unclear. In this study, we explored Lum’s different responses to drought stress in a comparison with Golden Promise (GP, drought-sensitive) and Tadmor (Tad, drought-tolerant). By analyzing the differences in the photosynthetic capacity, stomatal conductance, closure rate, and stomatal behavior of barley under drought stress, we found that the stomatal behaviors of Lum were significantly different from those of the drought-tolerant cultivar, Tad. A transcriptome analysis focused our attention on the ROS pathway, and further experiments revealed that this phenomenon could be ascribed to impaired ROS accumulation in Lum’s guard cells. These results suggest that different stomatal ROS responses affect stomatal behaviors, reflecting various drought regulation strategies in different barley varieties.

## 2. Materials and Methods

### 2.1. Plant Growth and Preparation

Golden Promise (GP, drought-sensitive), Lumley (Lum, drought-tolerant), and Tadmor (Tad, drought-tolerant) barley varieties were sowed in 1 L pots with potting mixture (mixed vermiculite and peat moss at a weight ratio of 9:1). Three healthy and uniform seedlings were maintained per pot. The plants were grown in a greenhouse on a 14-h/10-h day/night cycle (400 μmol m^−2^ s^−1^) and at 60% humidity. All plants were well watered before drought treatments, which commenced 3 weeks after sowing. From the fourth week of growth, drought stress was initiated by ceasing watering until the water-holding capacity decreased to ~5% (*v*/*v*), as measured by an HH2 soil moisture meter (Delta-T, England, UK). During the two-week drying experiments, the soil water content was maintained at ~5% by adding a moderate amount of water and recording the amount of water added to the calculation of the WUE. the control plants were continuously watered.

### 2.2. Water Deficit Drought Assay

Leaves detached from well-watered, four-week-old plants were dehydrated under laboratory conditions and weighed using a microbalance at 30 min intervals. Water loss was represented as (initial fresh weight—fresh weight at each time point)/initial fresh weight. The fresh weight of the aboveground part of each plant was determined on a microbalance in laboratory conditions. The dry weight was determined after drying at 65 °C for 3 days. The leaf relative water content (RWC) was calculated as (initial fresh weight − dry weight)/initial fresh weight.

### 2.3. Stomatal Assay

Stomatal apertures were measured on the epidermis of newly expanded leaves. Peels were taken from the abaxial leaf surface and fixed to the bottom of the laboratory glass after applying an optically clear and pre-pressure sensitive silicone adhesive to the laboratory surface. Peels were preincubated in opening buffer (5 mM MES-KOH, pH 6.1, and 50 mM KCl) for 1 h under 150 μmol m^−2^ s^−1^ photosynthetically active radiation (PAR) to open stomata. The peels were then imaged in the measuring buffer (5 mM MES-KOH, pH 6.1, and 10 mM KCl) for 20 min as the control, using a microscope (Nikon) equipped with a model No. NIS-F1 CCD camera and a DS-U3 controller (both Nikon). Images were taken every 5 min, and stomatal apertures were measured offline using ImageJ, version 1.51 (National Institutes of Health, Bethesda, MD, USA).

#### 2.3.1. ABA Treatment

In experiments with ABA (BBI LIFE SCIENCES, Shanghai, China), the measuring buffer was replaced with the ABA treatment solution (measuring buffer containing 0.1 mM ABA). The peels were measured for another 40 min after treatment and imaged to record the dynamic stomata closure.

#### 2.3.2. H_2_O_2_ Treatment

For experiments with H_2_O_2_ (SCR, Shanghai, China), the H_2_O_2_ treatment solution (measuring buffer containing 0.1 mM H_2_O_2_) was used instead of the measuring buffer. After treatment, the peels were measured for another 40 min to observe stomatal responses.

#### 2.3.3. CaCl_2_ Treatment

For experiments with CaCl_2_ (SCR, Shanghai, China), the measuring buffer was replaced with the CaCl_2_ treatment solution (measuring buffer containing 10 mM CaCl_2_). The peels were measured for another 40 min after treatment, and stomatal changes were recorded.

### 2.4. Gas Exchange Measurement

The youngest fully expanded leaves from four- to five-week-old plants were used for photosynthesis measurements using a portable gas exchange system (LI-6800, LI-COR Biosciences, Lincoln, NE, USA). The leaf chamber was maintained at a 400 μmol mol^–1^ CO_2_ concentration (*C_a_*), a leaf temperature of 25 °C, and an RH of 60%. Steady-state photosynthetic parameters were measured at 1400 μmol m^−2^ s^−1^ PAR. The value of stomatal limitation (*L_S_*) was calculated according to the formula proposed by Berry et al.: *L_S_* = 1 − *C_i_*/*C_a_* [52]. The non-stomatal limitation (*L_nS_*) was determined as: *L_nS_* = *C_i_*/*g_s_* [53,54]. The conditions were the same for light response curves, and after the initial 30 min of high-light adaptation, the light intensity (2000, 1800, 1600, 1400, 1200, 1000, 800, 600, 400, 200, 150, 100, 80, 50, 30, 15, and 0 μmol m^−2^ s^−1^ PAR) was decreased in steps of more than 3 min duration (stable state). For the CO_2_ response curves, the leaves were first acclimated at 400 μmol mol^−1^ *C_a_* under 500 μmol m^−2^ s^−1^ PAR, and the *C_a_* (400, 300, 200, 150, 100, 75, 50, 0, 400, 400, 600, 800, 1000, 1200, 1400, 1600, 1800, and 2000 μmol mol^−1^) was then changed stepwise at intervals of 3 min. To evaluate the potential stomatal kinetics of leaves in a light–dark transition, the conditions were the same as above, and the light intensity was alternately switched between light (1400 μmol m^−2^ s^−1^ PAR) and dark at 40 min and 100 min. The *A* and *g_s_* were recorded per min to analyze the halftime (t_1/2_) for stomatal opening and closure.

### 2.5. WUE Measurement

The *A* and *g_s_* of steady-state photosynthetic parameters, measured at a temperature of 25 °C, RH of 60%, 1400 μmol m^−2^ s^−1^ PAR, and 400 μmol mol^–1^ *C_a_*, were used for calculating the iWUE: iWUE = *A*/*g_s_*. Water consumption was recorded during the experiment. The WUE of the whole plant was determined as: WUE = dry weight (g)/water consumption (kg).

### 2.6. ROS Measurement

The production of ROS was monitored using the ROS-sensitive fluorescent probe H_2_DCFDA (Invitrogen, Waltham, MA, USA), as described by Wang et al. [15]. The epidermis was pre-treated with opening buffer for 1 h under light, then overlaid with 10 μM H_2_DCFDA in the same buffer for 20 min in darkness. Thereafter, peels were superfused with the measuring buffer to remove excess dye. The peels were incubated in measuring buffer with and without 100 μM ABA for 30 min, and the H_2_DCFDA fluorescence was monitored every 15 min using an Olympus FV3000 confocal microscope (Olympus, Tokyo, Japan). The H_2_DCFDA was excited by the 488 nm line, and the fluorescence emission was collected through a 505 to 550 nm bandpass filter. The background fluorescence recorded prior to H_2_DCFDA loading was corrected.

### 2.7. Antioxidant Capacity Measurement

Following the method of Wu et al. [55], fresh leaves were used to measure the superoxide dismutase (SOD), peroxidase (POD), and catalase (CAT) activities and malondialdehyde (MDA) contents, which were detected via micro colorimetric assays using Assay Kits (Sangon Biotech, Shanghai, China). The details of each measurement are described as follows:

#### 2.7.1. SOD Activity

Fresh tissue (0.1 g) from barley plants was homogenized with 1 mL extracting solution. The mixture was then centrifuged at 8000× *g* and 4 °C for 10 min to obtain the supernatant as a crude enzyme extract. The reaction solution (200 μL) included: 20 μL crude enzyme extract, 45 μL reagent I, 20 μL reagent II, 35 μL reagent III, 70 μL ddH_2_O, and 10 μL reagent IV. The absorbance at 560 nm was measured after the reaction solution was heated at 37 °C for 30 min. The SOD activity was expressed on a fresh weight basis as U g^−1^ FW.

#### 2.7.2. POD Activity

The POD activity was expressed as the A470 change per minute per gram of tissue in each mL. The reaction solution (245 μL) included: 120 μL reagent I, 30 μL reagent II, 30 μL reagent III, 60 μL ddH_2_O, and 5 μL crude enzyme extract. The absorption value was measured at 470 nm after the oscillator was fully mixed. The POD activity was expressed on a fresh weight basis as U g^−1^ FW.

#### 2.7.3. CAT Activity

The CAT activity was determined using the ultraviolet absorption method: 10 μL crude enzyme extract was added to 190 μL measuring solution (5 mL reagent I, +25 μL reagent II), shaken well, and measured at 240 nm. The CAT activity was expressed on a fresh weight basis as U g^−1^ FW.

#### 2.7.4. MDA Content

To measure the MDA content, 100 μL crude enzyme extract and 100 μL reagent III were added to 300 μL measuring solution (reagent II dissolved in 20 mL reagent I), shaken well, and then heated at 100 °C for 60 min. After rapid cooling, the mixture was centrifuged at 10,000× *g* for 10 min. The absorbance of the supernatant was measured at 532 nm and 600 nm. The MDA content was expressed on a fresh weight basis as nmol g^−1^ FW.

### 2.8. Transcriptome Analysis

After 21 days of drought treatment, the leaves were sampled for transcriptome analysis. An RNA sequencing library was constructed, and transcriptome sequencing and data analysis were performed on the Illumina HiSeq platform (OmicStudio, Hangzhou, China). A differential expression analysis of the two treatments was performed using the DESeq2 package, and the definition of the differential expression genes (DEGs) was as follows: fold change (drought vs. control) is log_2_N, |fold change| ≥ 1 and *p*-value < 0.05.

To confirm the reliability of the transcriptome data, RNA samples, as described above, were reversed using the PrimeScript™ II 1st strand cDNA synthesis kit, and a qRT-PCR was performed following the instructions of the TB Green Premix Ex Taq™ II (Both Takara, Japan). The qRT-PCR reaction was performed on a Light Cycler 480 System (Roche, Germany). The final values were averaged from three independent biological replicates. The sequences of the primers for 9 genes are listed in Table S1.

### 2.9. Data Analysis

Statistical analyses were carried out using SigmaPlot, version 14.0 (Systat Software; http://www.sigmaplot.com) (accessed on 22 March 2023). A significance was tested using an ANOVA and Duncan’s test. Otherwise, data are reported as means ± se of *n* observations. A bioinformatic analysis was performed using TBtools software [56] with a log scale and the online tools OmicStudio (https://www.omicstudio.cn/tool) (accessed on 13 October 2022). 

## 3. Results

### 3.1. Lum Exhibits a Strong Tolerance to Long-Term, Progressive Drought Stress

**Figure 1:**
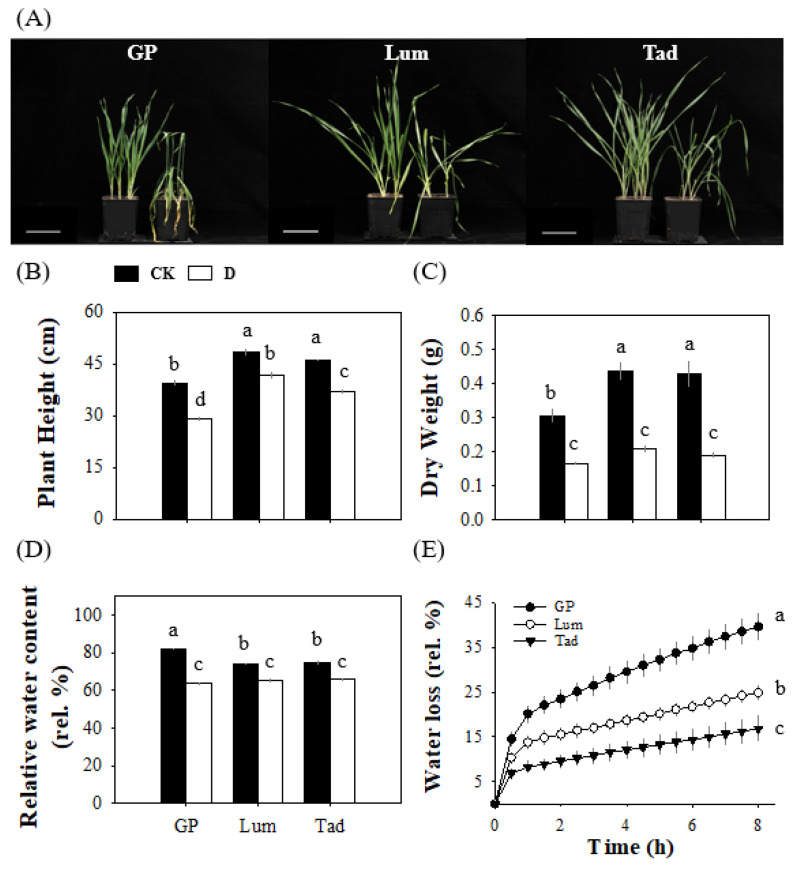
Different responses in three barley accessions under drought stress. (**A**) Drought performance of GP, Lum, and Tad after watering was ceased for 21 days. Scale bar: 5 cm. (**B**) Plant height, (**C**) dry weight (above ground), and (**D**) relative water content (RWC) in the leaves of GP, Lum, and Tad under control (CK, black bar) and drought (D, white bar) treatments. (**E**) Water loss in the detached leaves of GP, Lum, and Tad. Data are presented as means ± se (*n* = 3–5). Different letters above the error bars indicate significant differences at *p* < 0.05.

After 21 days of drought treatment, the growth of all varieties was severely affected by the water deficit (Figure 1A). Under drought stress, Lum showed an average reduction of 13.7 ± 1.9% in plant height compared to the control, while the plant heights of GP and Tad decreased by 26.2 ± 1.5% and 19.6 ± 1.4%, respectively (Figure 1B). In contrast, there was no significant difference in the dry weight among the three barley varieties with drought stress, despite the initial dry weight of Lum and Tad being greater than GP under control conditions (Figure 1C). Moreover, drought treatments led to a dramatic reduction in the leaf relative water content (RWC) in all three cultivars (GP, Lum, and Tad: 22.3 ± 1.0%, 11.5 ± 1.2%, and 12.1 ± 1.1%, respectively), especially GP (Figure 1D), indicating that Lum and Tad demonstrate better tolerance than GP. The leaf water loss rate reflects the plant’s water-holding capacity and drought resistance. Therefore, we measured the rate of water loss from the isolated leaves of three barley cultivars. As shown in Figure 1E, the water loss of the tolerant genotype, Lum, was significantly lower than that of the drought-sensitive genotype, GP, and that of Tad was even significantly lower than Lum (Figure 1E). Maintaining water content and minimizing water loss is an effective way for plants to survive under drought conditions. In plants, roughly 90% of water loss occurs through stomata [57]. Based on these results, we speculated that the various drought responses shown in these three barley genotypes may well be due to their different stomatal responses to water deficit.

### 3.2. Lum Maintains Higher Stomatal Conductance for Photosynthesis under Drought Stress

**Figure 2:**
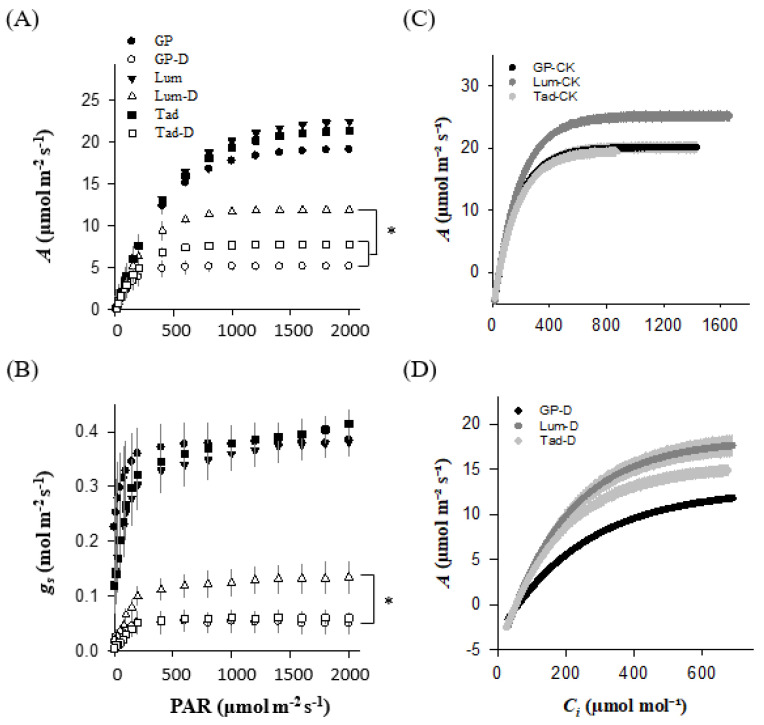
Photosynthetic curves for GP, Lum, and Tad. (**A**,**B**) Net photosynthesis (*A*) and stomatal conductance (*g_s_*) were measured by the light response curves, generated at a temperature of 25 °C, RH of 60%, and 400 μmol mol^–1^ C_a_. (**C**,**D**) CO_2_ response curves were generated at a temperature of 25 °C, RH of 60%, and 2000 μmol m^−2^ s^−1^ PAR. Data are presented as the means ± se (*n* = 3–4). * indicates significant differences at *p* < 0.05.

Gas exchange measurements were carried out in the three species, and the light response and CO_2_ response curves are presented in Figure 2. No significant difference in the light response curves was observed under control conditions (Figure 2A). However, there was a significant difference in the net photosynthesis (*A*) in the drought-treated groups when the photosynthetically active radiation (PAR) was over 50 μmol m^−2^ s^−1^ (Figure 2A). The maximum light-saturated photosynthetic rate (*A*_Lmax_) was the highest in the control group for Lum (24.48 ± 0.18 μmol m^−2^ s^−1^), followed by Tad (22.70 ± 0.42 μmol m^−2^ s^−1^). The *A*_Lmax_ was the lowest for GP (17.35 ± 2.43 μmol m^−2^ s^−1^). The *A*_Lmax_ values of GP and Tad in the treatment groups were significantly lower than those of Lum, reaching only approximately 43.46 ± 9.27% and 64.94 ± 8.62% of Lum, respectively (Table S2). The light saturation point (LSP) was higher in Lum (1734.27 ± 363.66 μmol m^−2^ s^−1^) than GP and Tad (993.69 ± 30.04 and 1256.95 ± 185.19 μmol m^−2^ s^−1^, respectively) after drought treatments, but there were no differences in the control groups; however, the apparent quantum yield (AQY) and light compensation point (LCP) were similar among the three species (Table S2). This suggests that Lum has greater *A* than Tad and GP under drought conditions.

After strong light induction (over 400 μmol m^−2^ s^−1^ PAR), the *g_s_* of the Lum in the treatment group was significantly higher than that of the GP and Tad (Figure 2B). However, no difference was observed under control conditions (Figure 2B). Stomatal conductance (*g_s_*) regulation is considered a major mechanism responsible for regulating plant responses to water stress, since stomatal closure is one of the earliest responses to water shortage and a major determinant of limitation to photosynthesis [58]. It was speculated that this may be one of the important reasons why Lum was capable of maintaining a higher *A* under drought stress.

Lum also had higher *A* values than GP and Tad at different intracellular CO_2_ concentrations (*C_i_*) in both the control and drought-treated groups (Figure 2C,D and Table S2). In the control group, the maximum CO_2_-saturated photosynthetic rate (*A*_Cmax_) observed for Lum (24.69 ± 1.09 μmol m^−2^ s^−1^) was significantly higher than GP and Tad (20.10 ± 0.88 and 19.41 ± 0.62 μmol m^−2^ s^−1^, respectively). For all cultivars, the net *A*_C_ decreased under drought stress. The *A*_Cmax_ values of the Lum and Tad (17.18 ± 1.32 and 14.28 ± 0.80 μmol m^−2^ s^−1^, respectively) in the treatment groups were significantly higher than those of the GP (10.18 ± 1.53 μmol m^−2^ s^−1^) (Table S2). Among the above, Lum demonstrated a better CO_2_ utilization capacity than GP and Tad under the control and drought conditions.

The apparent CO_2_ compensation point, calculated from the *A*/*C*_i_ curves, was significantly lower in Lum and Tad (51.57 ± 0.74 and 52.54 ± 0.84 μmol, respectively) compared with GP (63.92 ± 4.94 μmol) under drought stress (Table S2). However, no significant difference was found in the control groups, indicating an improved CO_2_ refixation in these drought-tolerance cultivars [59]. The maximum rates of ribulose-1,5-bisphosphate (RuBP) carboxylase/oxygenase (Rubisco) carboxylation (*V*_cmax_), electron transport driving the regeneration of RuBP (*J*_max_), and triose-phosphate utilization (*V*_TPU_) are now widely assumed to represent major limitations to light-saturated photosynthesis. Typically, *V*_TPU_ will not be a limitation at any *C_i_*. Therefore, only two phases may be seen and noticed [60]. As is shown in Table S2, the *V*_cmax_ in the control group was higher in Lum and Tad (38.01 ± 2.26 and 39.48 ± 1.14 μmol m^−2^ s^−1^, respectively) than GP (32.09 ± 2.59 μmol m^−2^ s^−1^), while under drought stress, the *V*_cmax_ was significantly higher in Lum (44.37 ± 1.08 μmol m^−2^ s^−1^) than in GP and Tad (32.91 ± 1.35 and 36.39 ± 1.44 μmol m^−2^ s^−1^, respectively). Under control conditions, *J*_max_, which represents the RuBP regeneration capacity [60], was higher in Lum (43.77 ± 2.74 μmol m^−2^ s^−1^) and Tad (47.28 ± 0.77 μmol m^−2^ s^−1^) when compared with GP (37.57 ± 2.99 μmol m^−2^ s^−1^). Drought stress increased the *J*_max_ of Lum to 58.25 ± 0.49 μmol m^−2^ s^−1^, significantly higher than GP (42.24 ± 3.53 μmol m^−2^ s^−1^) and Tad (43.72 ± 1.31 μmol m^−2^ s^−1^). Therefore, it appears that the availability of CO_2_ under drought limits the *A* of GP, which may be due to the smaller *g_s_*.

**Figure 3:**
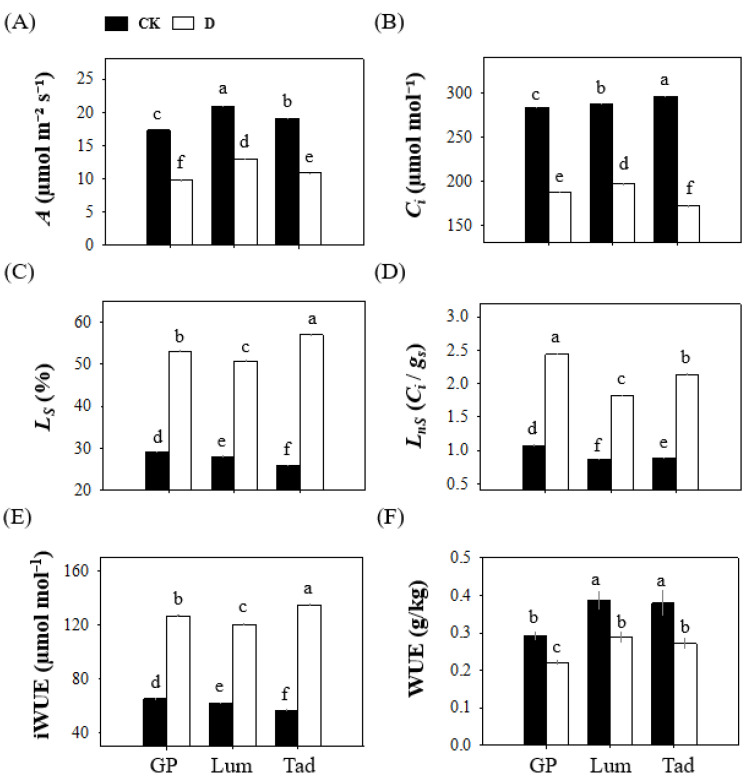
Photosynthetic parameters for GP, Lum, and Tad. Steady-state *A* (**A**), *C_i_* (**B**), *L_S_* (**C**), *L_nS_* (**D**), and iWUE (**E**) measured at a temperature of 25 °C, RH of 60%, 1400 μmol m^−2^ s^−1^ PAR, and 400 μmol mol^–1^ *C_a_*. *L_S_* = 1 − *C_i_*/*C_a_*; *L_nS_* = *C_i_*/*g_s_*; iWUE = *A*/*g_s_*. (**F**) WUE = dry weight (g)/water consumption (kg). Data are presented as the means ± se (*n* = 3–4). Different letters above the error bars indicate significant differences at *p* < 0.05.

We then measured the steady-state photosynthetic parameters and analyzed the stomatal and non-stomatal limitations and the WUE (Figure 3). Factors leading to reduced photosynthesis can be divided into stomatal and non-stomatal limitations. Under drought stress, intercellular CO_2_ concentrations (*C_i_*) cannot meet the needs of photosynthesis due to the limitations of *g_s_*, known as stomatal limitations (*L_S_*) [61]. On the other hand, the photosynthesis capacity of the mesophyll is limited, showing a decrease in the activity of photosynthesis organs, i.e., non-stomatal limitations (*L_nS_*) [62,63]. Therefore, *A* reduction is mainly dependent on *L_S_* and *L_nS_*. The variation trends in the *C_i_* and *L_S_* values are two key factors that determine whether the *A* of crops is restricted by *L_S_* or *L_nS_*. From our results, the decreasing *C_i_* and increasing *L_S_* indicate that the *A* of all three varieties were mainly suffering from *L_S_* at this stage (Figure 3A–C). The higher *A* in Lum was most likely due to the high *g_s_* under the drought condition. We then analyzed the WUE at both the leaf- and whole plant levels. At the leaf level, the iWUE increased in all three varieties after drought treatment (Figure 3E). However, the iWUE of Lum (120.29 ± 0.39 μmol mol^−1^) was significantly lower than that of GP and Tad (126.93 ± 0.54 and 134.99 ± 0.42 μmol mol^−1^, respectively) under drought stress, reflecting different changes in *g_s_* and *A*. Interestingly, the WUE of both Lum and Tad was higher than GP under control and drought conditions (Figure 3F). This result suggests that the whole plant WUE is influenced by multiple factors. For example, the leaf cuticle membranes of Lum and Tad were significantly thicker than GP (Figure S3), preventing water loss from the mesophyll cells.

In summary, Lum has higher *A*, *g_s_*, and WUE values under drought. The *A*/*C_i_* curve also indicates that Lum has a higher *A* at the same *C_i_* compared to GP. We suggest that Lum could maintain an increased *g_s_* and increase CO_2_ utilization, thus improving *A* and allowing it to grow better under drought stress.

### 3.3. Lum Exhibits a Sluggish Stomatal Closing under Drought Condition

**Figure 4:**
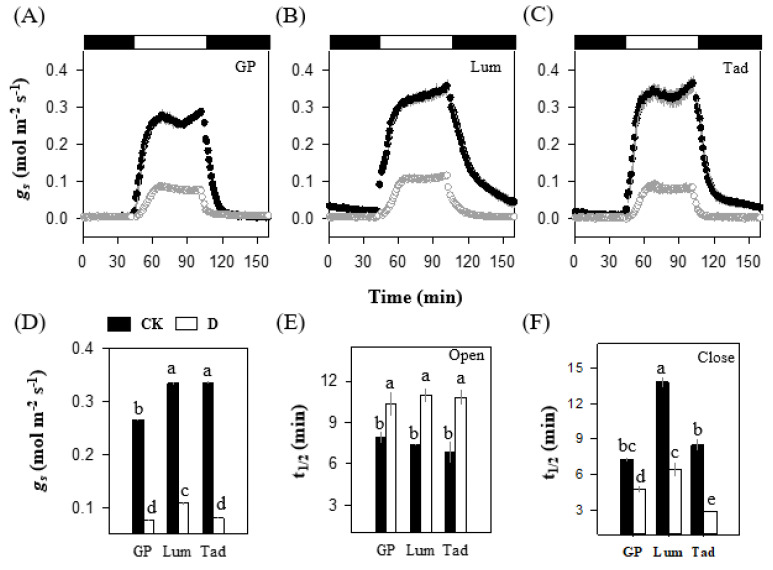
Dynamics of stomatal conductance (*g_s_*) and time constants for stomatal kinetics under light–dark transition. (**A**–**C**) Dynamics of *g_s_* at the leaf level in controlled light environments in three species. The light intensity was alternately switched between light (1400 μmol m^−2^ s^−1^ PAR, white bar) and dark (black bar) at 40 min and 100 min. (**D**) Steady-state *g_s_*_max_ during the high-light (1400 μmol m^−2^ s^−1^ PAR, white bar) period. (**E**,**F**) Halftime for stomatal opening (t_1/2open_) and closing (t_1/2close_) determined by photosynthesis measurements. Data are presented as the means ± se (*n* = 3–4). Different letters above the error bars indicate significant differences at *p* < 0.05.

We then measured the dynamics of *g_s_* to study stomatal responses to the light–dark switching among the three species. The dynamics of the leaf-level photosynthetic characteristics, *g_s_*, were similar among three species, reaching *g_s_*_max_ during the high-light period and dropping during the dark period. However, the stomatal responses were clearly different among the three species (Figure 4A–C). In the control group, the *g_s_*_max_ was significantly higher in Lum and Tad than in GP, while in the drought group, a higher *g_s_*_max_ was observed in Lum than in GP and Tad (Figure 4D). A quantitative analysis of leaf-level stomatal dynamics showed that the t_1/2open_ was similar among the three varieties under the same treatment (Figure 4E). On the contrary, the t_1/2close_ in Lum were markedly larger when compared to GP and Tad. Both in the control and drought-treated groups, the t_1/2close_ were the largest in Lum (13.76 ± 0.43 min and 6.46 ± 0.55 min, respectively) (Figure 4F and Table S3), demonstrating a slow change in *g_s_* in response to the start of dark period, whereas GP and Tad showed a rapid change in *g_s_* and a significant difference in t_1/2close_ under drought stress (7.27 ± 0.11 min and 8.50 ± 0.49 min in the control group and 4.78 ± 0.19 min and 2.97 ± 0.03 min in the drought-treated group, respectively) (Figure 4F and Table S3). Based on the dynamic stomatal conductance results, Lum exhibited a sluggish stomatal closing compared to Tad.

### 3.4. ABA-Induced Stomatal Response Is Impaired in Lum

**Figure 5:**
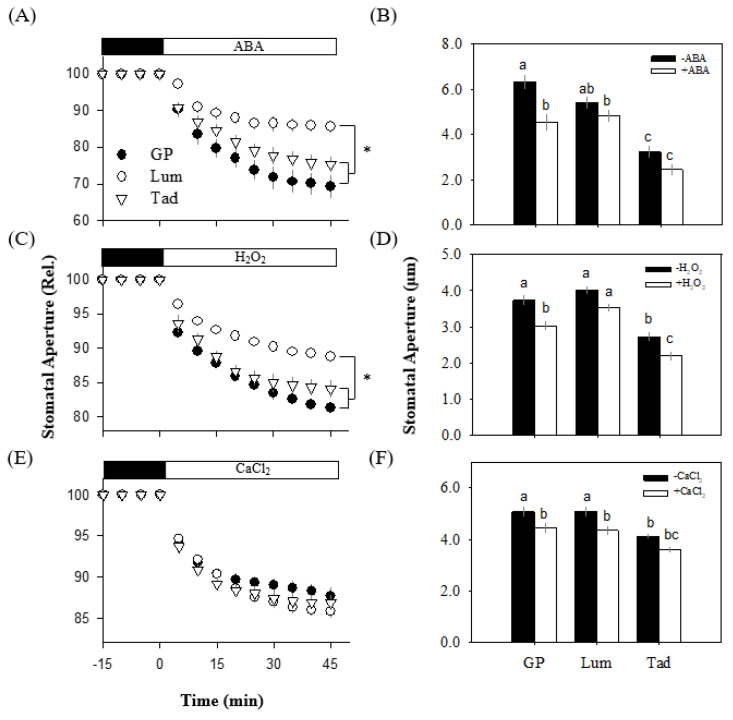
ABA-, H_2_O_2_- and CaCl_2_-induced stomatal closure in barley. Stomatal closure and parallel aperture induced by adding 100 μM ABA (**A**,**B**), 100 μM H_2_O_2_ (**C**,**D**), and 10 mM CaCl_2_ (**E**,**F**) in GP (black circles), Lum (white circles), and Tad (white triangles) guard cells. Apertures were normalized on a cell-by-cell basis to values at a time point 15 min before treatments. Stomata were pooled from four independent experiments for each line. Data are presented as the means ± se (*n* = 30—40). Different letters above the error bars and * indicate significant differences at *p* < 0.05.

As Lum exhibited a sluggish stomatal closing under drought conditions, we examined its stomatal response to ABA. In epidermal peels of GP, Lum, and Tad plants, most stomata were open during the day in a growth chamber (Figure 5). We applied exogenous ABA to monitor the dynamics of stomatal aperture changes, as shown in Figure 5A, for the three species after 45 min of ABA treatment. The stomatal aperture closed rapidly within the first 10 min after ABA addition, and the rate of aperture change decreased after 10 min. ABA-induced stomatal closure was consistent in the epidermal peels of both GP and Tad, while the stomata of Lum showed only a slight decrease in the measuring buffer for a period of 45 min under the microscopic light, and the relative stomatal aperture of Lum (85.58 ± 1.70%) was significantly higher than that of GP and Tad (69.28 ± 3.27% and 75.37 ± 2.09%, respectively) (Figure 5A,B).

ABA elevates [Ca^2+^]_i_ by coordinating Ca^2+^ entry at the plasma membrane with Ca^2+^ release from endomembrane stores, a process often described as Ca^2+^-induced Ca^2+^ release [64,65]. ABA activates plasma membrane Ca^2+^ channels [66], facilitating Ca^2+^ influx [65,67,68]. The rise in [Ca^2+^]_i_ is partly related to ROS [22]. Thus, we measured stomatal responses to exogenous H_2_O_2_ and CaCl_2._ The application of 100 μM H_2_O_2_ revealed that the overall results were similar to the ABA induction results, and the relative stomatal aperture of Lum (88.84 ± 0.54%) was significantly higher than the relative stomatal apertures of GP and Tad (81.38 ± 0.84% and 84.12 ± 1.35%, respectively), although the differences were reduced (Figure 5C,D). Interestingly, stomatal sensitivity was also tested with 10 mM CaCl_2_ (commonly used to trigger Ca^2+^-induced stomatal closure), and no significant difference in stomatal response was demonstrated among GP, Lum, and Tad (87.66 ± 0.74%, 85.79 ± 0.69% and 86.87 ± 0.87%, respectively) (Figure 5E,F). Thus, we speculated that the difference in stomatal response to ABA between Lum and the other two species was due to the production of ROS upstream of the Ca^2+^ signal.

### 3.5. Transcriptome Analysis Revealed Changes in ROS-Associated Genes in the Drought-Tolerant Genotype Lum

**Figure 6:**
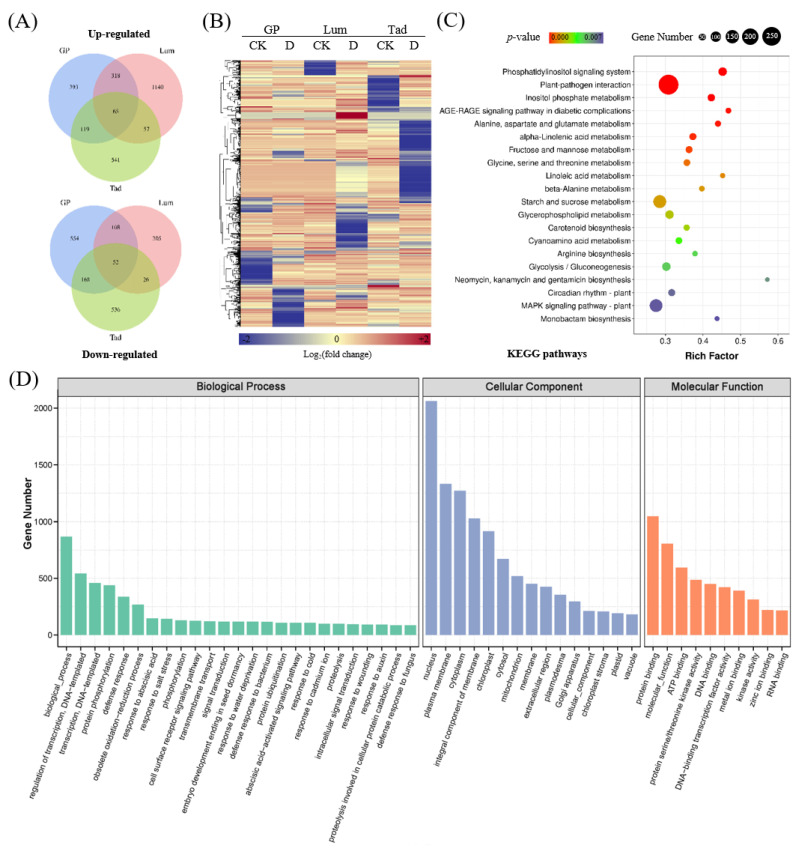
Identification and characterization of drought-responsive, differentially expressed genes (DEGs) in GP, Lum, and Tad. (**A**) Venn diagram of up- and downregulated DEGs in three barley genotypes. (**B**) Hierarchical clustering analysis of drought-responsive DEGs. The samples and treatments are shown under each column. (**C**) Enrichment analysis of drought-responsive DEGs by the Kyoto Encyclopedia of Genes and Genomes (KEGG). The color of the dot represents *p*-value, and the size of the dot represents the number of DEGs mapped to the reference pathway. (**D**) Gene ontology (GO) analysis of drought-responsive DEGs. GO terms are divided into cellular component, molecular function, and biological process, respectively.

In order to further determine the differences in the molecular mechanisms of drought tolerance in GP, Lum, and Tad, an RNA-seq was performed after 21 days of drought treatment. To validate the transcriptome data, a qRT-PCR was performed. The trends in the expression patterns of the selected genes by qRT-PCR were consistent with those identified by the transcriptome, indicating that the RNA-seq assay was reliable (Figure S1). We further analyzed the differentially expressed genes (DEGs) with Venn. After 21 days of drought treatment, 895, 1580, and 782 genes were upregulated and 885, 491, and 782 genes were downregulated in GP, Lum, and Tad, respectively (Figure 6A). Here, we focused on the DEGs with an upregulated expression in Lum and a downregulated or un-regulated expression in GP and Tad, unregulated expression in Lum and downregulated expression in GP and Tad, or up/downregulated expression in the three genotypes as general response genes. These genes may play key roles in drought tolerance. A total of 1419 drought-tolerance-associated genes were identified according to different expression patterns in GP, Lum, and Tad (Figure 6B). A KEGG enrichment analysis showed that most of these genes were related to plant–pathogen interactions; alanine, aspartate and glutamate metabolism, carotenoid biosynthesis; glycolysis/gluconeogenesis; and the MAPK signaling pathway (Figure 6C). A GO analysis revealed significant enrichment in catalytic activity, binding activity, metabolic processes, cellular components, cellular processes, biological regulation, response to stimulation, and developmental processes (Figure 6D). Combined with gene annotation, we found that 20 significantly differentially expressed genes were mainly involved in stomatal regulation, MAPK signaling, photosynthesis, and Ca^2+^-transport (Table S4). Based on earlier results, we focused on ROS-related genes and performed a sequence alignment based on *Arabidopsis* ROS-scavenging network genes [69]. We found at least 112 homologous genes in barley (Figure S2), 24 of which are listed in Table 1. ROS genes were indeed involved in the drought response. As shown in Table 1, eight genes were significantly upregulated in Lum and unregulated or significantly downregulated in GP and Tad, and the regulatory trend of the other fourteen genes in Lum was opposite to the trend in GP and Tad (Table 1). These results suggest that these pathways influence drought tolerance in plants by influencing the expression pattern of these genes, further indicating the potential role of ROS in Lum for different stomatal responses.

### 3.6. ABA-Induced ROS Accumulation Was Impaired in Lum

ROS are essential secondary messengers and signaling molecules in various plant responses to environmental conditions [70]. ABA is known to stimulate the synthesis of ROS in guard cells which, in turn, helps to activate Ca^2+^ channels and trigger Ca^2+^-dependent signal cascades [19,22]. Together with the stomatal assay and transcriptome results, we inferred that the difference in the stomatal response to ABA between Lum and the other two species was due to the production of ROS upstream of the Ca^2+^ signal.

**Figure 7:**
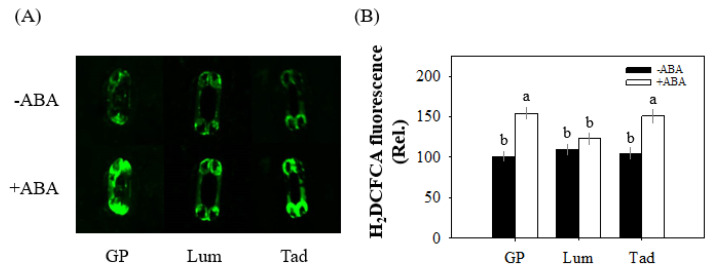
Guard cell reactive oxygen species production in response to ABA. (**A**) The images show representative H_2_DCFDA fluorescence of guard cell pairs. (**B**) ROS level measured using H_2_DCFDA fluorescence in guard cells of the GP, Lum, and Tad plants after 30 min either without or with 100 μM ABA. Data are presented as the means ± se (*n* = 20–30). Different letters above the error bars indicate significant differences at *p* < 0.05.

Therefore, we next examined whether ROS production was suppressed in the guard cells of Lum. The guard cells were loaded by incubation [71] with the fluorescent dye 2,7-dichlorofluorescein diacetate (H_2_DCFDA), which reported the total ROS activity in the cell [72,73]; this is uncommon in previously published work in barley. Figure 7 summarizes the results of four independent experiments and measurements for each line. We found that H_2_DCFDA fluorescence increased roughly 1.5-fold and 1.4-fold, respectively, after 30 min of treatment of the GP and Tad guard cells with 100 μM ABA, suggesting a significant increase in ROS production. At the same time, the guard cells of Lum showed only a 1.1-fold rise in H_2_DCFDA fluorescence (Figure 7B), indicating that ABA-induced ROS accumulation was impaired in Lum.

### 3.7. Different Antioxidant Activities of Three Varieties Reflect Different Response Patterns

In plant cells, the enzymatic antioxidant system against ROS includes the continuous and simultaneous action of multiple enzymes (such as SOD, POD, and CAT), which induce oxidative stress due to exposure to abiotic or biotic stresses [74,75,76]. MDA can be used as an indicator of the degree of peroxidation and can indirectly determine the degree of damage to plant membrane systems and resistance to stress [77].

**Figure 8:**
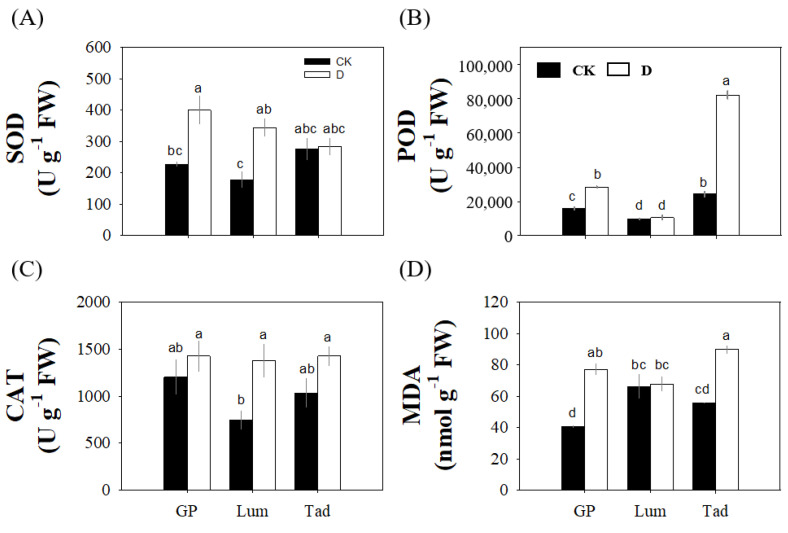
Antioxidant capacity assessment in GP, Lum, and Tad. Activities of SOD (**A**), POD (**B**), CAT (**C**), and contents of MDA (**D**) under control (CK, black bar) and drought (D, white bar) stress conditions. Data are presented as the means ± se of three biological replicates and different letters above the error bars indicate significant differences at *p* < 0.05.

Therefore, we tested the plants’ antioxidant capacity by measuring the enzyme activities of SOD, POD, and CAT and the production of MDA in GP, Lum, and Tad (Figure 8). We found that drought stress resulted in an increase in the SOD activities of GP and Lum (Figure 8A). Although the SOD activity did not change significantly, Tad showed higher SOD activities in both the control and drought conditions (Figure 8A). The POD activities were significantly higher in Tad than GP and Lum after drought treatment (Figure 8B). Drought stress showed little effect on POD activities in Lum, but Lum showed significantly higher drought-induced increases in CAT activities compared to GP and Tad (Figure 8C). The MDA content in GP and Tad was increased by 89.46 ± 8.40% and 61.36 ± 4.66% under drought stress when compared to the control, which showed little change in Lum (Figure 8D).

In conclusion, under drought stress, Lum did demonstrate higher SOD and CAT activities, GP showed higher SOD activities and MDA content, and Tad showed higher POD activities and MDA content. They seem to rely differently on these different types of antioxidant activities, reflecting different response patterns and contributing to non-stomatal limitations.

## 4. Discussion

**Figure 9:**
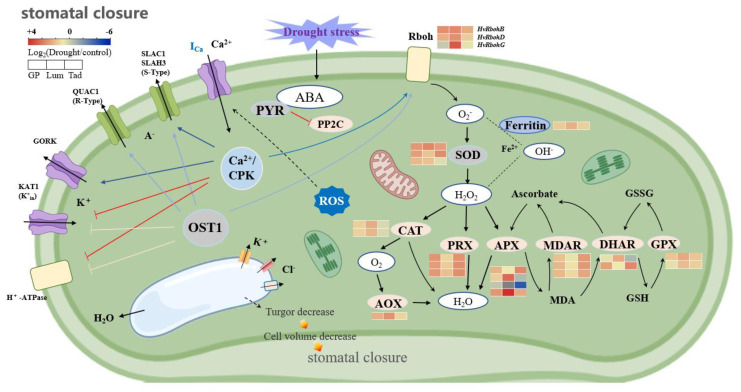
A hypothetical working model of underlying stomatal closure mechanisms in Lum. The relative expression levels of genes encoding different enzymes are shown by a color gradient from low (blue) to high (red). For each heatmap, from left to right: GP (first column), Lum (second column), Tad (third column). Rboh—NADPH oxidase enzyme; PYR—ABA receptors; PP2Cs—2C-type protein phosphatases; SOD—superoxide dismutase; APX—ascorbate peroxidase; CAT—catalase; GPX—glutathione peroxidase; PRX—peroxiredoxin; AOX—Alternative oxidase; MDAR—monodehydroascorbate reductase; DHAR—dehydroascorbate reductase; OST1—OPEN STOMATA 1; I_Ca_—calcium channels; SLAC1 and SLAH3—S-type anion channels; QUAC1—R-type anion channel; KAT1—K^+^_in_ channel; GORK—guard cell outward rectifying K^+^ channel; CPK—calcium-dependent protein kinase.

Drought is the most severe environmental stress, reducing crop yield and quality worldwide by affecting various physiological and biochemical processes within plants. With an increasing pressure to improve crop productivity, stomata are central to improving photosynthesis and water use, as well as unexplored targets to improve both processes and the balance between them [35,37,78]. Stomata regulate the water exchange between plants and the outside world and play an important role in plant response to drought. Compared to the kidney-type stomatal structure of dicotyledons, the stomatal structure and developmental peculiarities of *Poaceae* crops, which consist of two guard cells and two secondary guard cells, have great potential in improving water utilization in crops [79,80]. In-depth studies on the behavioral differences of stomata in response to drought stress in barley and other grain crops provide some theoretical guidance for crop breeding for drought tolerance. In the present study, we found that Lum (drought-tolerant) and Tad (drought-tolerant) demonstrated less water loss and better WUE than GP (drought-sensitive) (Figure 1 and Figure 3). Light response and CO_2_ response curves suggested that Lum exhibited greater CO_2_ assimilation during the growth period, achieved by a high *g_s_* (Figure 2), whereas Lum plants exhibited substantially slower stomatal closure than Tad plants in response to the light–dark transition (Figure 4). The reason why Lum demonstrated a strong drought resistance but a slow stomatal close attracted our attention. Therefore, we further explored the physiological impact of ABA, H_2_O_2_, and CaCl_2_ on stomatal closure in the three species (Figure 5) and found that the difference in stomatal response between Lum and the other two species may be due to ROS. Through a transcriptome analysis and ROS and enzyme activity measurements (Figure 6, Figure 7 and Figure 8), we concluded that different stomatal ROS responses affect stomatal closure, showing different drought regulation strategies. Based on these results, we proposed a predicted model of the mechanism involved in stomatal closure and drought tolerance (Figure 9).

Under drought stress, ABA acts as an endogenous anti-transpirant and activates a series of cellular signaling pathways that lead to the rapid closure of plant stomata to reduce the rate of water loss through stomatal pores in the leaf epidermis [13]. ROS are involved in the regulation of ABA-mediated stomatal signaling [22,47]. Organelles with a high oxidative metabolic activity or strong electron flow rates, such as chloroplasts, mitochondria, and microbodies, are the main sources of ROS production in plant cells. Together with a large number of oxidases, plant cells are sufficiently armed to produce large and flexible amounts of ROS [69]. A key player in the network of ROS-producing enzymes is the specialized respiratory burst, RBOH [51], which has been the subject of intense investigation. ABA can enhance gene expression [19] and NADPH oxidase activity [81]. It was reported that the two partially redundant *Arabidopsis* NADPH oxidase catalytic subunit genes *AtrbohD* and *AtrbohF* function in ABA signal transduction in guard cells [19]. Most forms of biotic or abiotic stress disrupt the metabolic balance of cells, resulting in enhanced production of ROS [69]. In this study, homologous genes of *RbohB*, *RbohD*, and *RbohG* were identified in barley. Under drought stress, *HvRbohG* (HORVU5Hr1G078630) was significantly upregulated in Lum, while it was significantly downregulated in GP and Tad. *HvRbohD* (HORVU3Hr1G069780) and *HvRbohB* (HORVU3Hr1G037600) were significantly upregulated in Lum and were less regulated in GP and Tad. We believe this is the reason, at least in part, for the differences in ROS production between the species under drought stress.

It is an integral feature of the cellular metabolism to prevent the excessive production or scavenging of ROS and maintain its normal metabolic level [82,83]. ROS can be generated by various enzymatic activities and can be removed by a series of ROS-scavenging enzymes. The main ROS-scavenging enzymes of plants include SOD, CAT, GPX, PRX, and ascorbate peroxidase (APX) (Figure 9). Ascorbic acid and glutathione are maintained in their reduced state by a group of enzymes such as monodehydroascorbate reductase (MDAR) and dehydroascorbate reductase (DHAR). These enzymes provide cells with an effective mechanism to detoxify O_2_^−^ and H_2_O_2_ in combination with the antioxidants ascorbic acid and glutathione [84]. The balance between SODs and the different H_2_O_2_-scavenging enzymes in cells is considered the key to determining the steady-state level of O_2_^−^ and H_2_O_2_. This balance, together with the sequestering of metal ions by ferritin and other metal-binding proteins, prevents the formation of the highly toxic HO· radical [69,82,85]. The coordination pattern between different components of the ROS-scavenging network of plants is complex. In our study, several genes that encode ROS-scavenging enzymes are shown in Figure 9. *HvAOX* (HORVU2Hr1G122660), *HvAPXs* (HORVU7Hr1G083550 and HORVU5Hr1G097270), *HvFerritin* (HORVU5Hr1G047730), and *HvSOD* (HORVU2Hr1G021110) were significantly upregulated in Lum but unregulated or downregulated in GP and Tad. *HvSODs* (HORVU7Hr1G060130 and HORVU7Hr1G008390), *HvCATs* (HORVU6Hr1G008640 and HORVU6Hr1G008730), *HvGPX* (HORVU2Hr1G096960), *HvPRXs* (HORVU2Hr1G073760, HORVU7Hr1G033500 and HORVU6Hr1G034620), *HvAPXs* (HORVU2Hr1G101730 and HORVU6Hr1G009500), *HvDHARs* (HORVU5Hr1G045850 and HORVU7Hr1G038770), *HvMDARs* (HORVU1Hr1G013740, HORVU2Hr1G023170, and HORVU7Hr1G101500), and *HvGPXs* (HORVU2Hr1G096960 and HORVU6Hr1G063830) were differentially expressed in Lum and the two other species. In addition, while its MDA content increased less (Figure 8), Lum exhibited significant increases in SOD and CAT activities in response to drought stress, indicating a different model of scavenging ROS.

Under drought conditions, guard cells elevate ABA levels, which activate OST1 and other signaling mechanisms, including molecules such as ROS and Ca^2+^. ROS are essential secondary messengers and signaling molecules in plant responses to environmental conditions [70]. ABA is known to stimulate ROS synthesis in guard cells which, in turn, contributes to the activation of Ca^2+^ channels and triggers Ca^2+^-dependent signal cascades [19,22]. OST1 acts upstream of ROS in ABA signaling in guard cells [86], and it cooperates with ROS, Ca^2+^, and Ca^2+^-dependent protein kinases to activate the S-type anion channel SLAC1 and other ion channels, Ca^2+^-permeable channels, K^+^ efflux channels, and RBOH and inhibit the H^+^ pumps and K^+^ influx channels. Ion efflux from guard cells is accompanied by the osmotic water efflux, resulting in the loss of guard cell turgor and stomatal closure. Stomatal closure reduces plant water loss during drought [19,87,88,89,90].

In the present study, we found that Lum is a drought-tolerant genotype, similar to Tad. However, compared to Tad, Lum demonstrated a higher net photosynthetic rate and stomatal conductance (Figure 1 and Figure 2), slower stomatal closure rate under drought stress (Figure 4), and less-sensitive ABA-induced stomatal closure (Figure 5). A transcriptome analysis indicated that ROS-related genes were indeed involved in drought response regulation and may be responsible for the difference in stomatal response between Lum and Tad (Figure 6). Further studies revealed that the slow stomatal closure rate in Lum could be attributed to impaired ROS accumulation in guard cells (Figure 7 and Figure 8). These results suggest that different stomatal reactive oxygen responses affect stomatal closure, resulting in different drought regulation strategies. The complex genetic background of the experimental materials posed a great challenge for us to study drought resistance. Stomatal conductance is closely related to WUE in plants [25,26,27,28]. The significantly higher stomatal closure rate of Tad than GP may contribute to a stronger drought resistance than GP. The nonsynchronous response between a rapid photosynthetic rate and a sluggish stomatal movement resulted in a lower iWUE of Lum under drought stress (Figure 3). However, the WUE of Lum was insignificantly different from Tad and was significantly higher than GP. This might be achieved through a reduction in the water loss of the whole plant by other means, such as its thicker cuticle (Figure S3). Most genes encoding ROS production and ROS-scavenging proteins were both significantly upregulated in Lum (Figure 9); this cannot directly explain the impaired ROS accumulation in Lum. The complex nature of the ROS gene network, its integration into the web of plant signaling networks, and the large plasticity in guard cell function demonstrate the difficulty in fully elucidating the specific mechanisms involved in stomatal behavior and responses to ROS signaling [37]. Fortunately, we have proposed a possible working model of stomatal response mechanisms that can be further tested through molecular biology in future work.

## 5. Conclusions

Physiological and transcriptome analyses revealed the stomatal responses of two drought-tolerant barley varieties, Lum and Tad, with different ROS regulation strategies under drought conditions. Compared to Tad, Lum showed a higher *A* and *g_s_*, slower stomatal closure rate under drought stress, less-sensitive ABA-induced stomatal closure, fewer drought-induced increases in ROS and MDA, and a dependence on different types of antioxidant activities. The ROS-related regulatory genes under drought stress were also identified. These findings enhance our contribution to understanding the effects of different ROS regulation strategies on stomatal response and provide a testable working model of stomatal response mechanisms for future work.

## Figures and Tables

**Table 1:** Main differentially expressed ROS genes in GP, Lum, and Tad under drought.

Group	AGI Code	Gene ID	Fold Change (Drought vs. Control)		
			GP	Lum	Tad
Alternative oxidase (AOX)	At4g22260.1	HORVU2Hr1G122660	0.31	1.00	−0.49
Ascorbate peroxidase (APX)	At4g09010.1	HORVU2Hr1G101730	0.27	−0.93	1.22
	At3g09640.1	HORVU5Hr1G097270	−0.36	1.89	−2.37
	At4g32320.1	HORVU6Hr1G009500	−2.00	-	−5.21
	At4g35970.1	HORVU7Hr1G083550	0.88	3.24	0.47
Catalase (CAT)	At1g20630.1	HORVU6Hr1G008640	−0.37	0.41	−1.50
	At4g35090.1	HORVU6Hr1G008730	−0.44	0.15	−1.50
Dehydroascorbate reductase (DHAR)	At5g16710.1	HORVU5Hr1G045850	−1.24	0.42	−2.42
	At5g16710.1	HORVU7Hr1G038770	0.62	−0.98	1.22
Ferritin	At5g01600.1	HORVU5Hr1G047730	0.44	2.91	0.10
Glutathione peroxidase (GPX)	At2g31570.1	HORVU6Hr1G063830	−0.17	0.46	−0.19
	At1g63460.1	HORVU2Hr1G096960	−1.25	0.05	−0.36
Monodehydroascorbate reductase (MDAR)	At5g03630.1	HORVU1Hr1G013740	−0.09	−1.18	0.82
	At3g09940.1	HORVU2Hr1G023170	0.14	−0.77	0.86
	At5g03630.1	HORVU7Hr1G101500	0.12	−0.58	0.51
NADPH oxidase (RBOH)	At1g09090.2	HORVU3Hr1G037600	1.05	1.18	0.38
	At5g47910.1	HORVU3Hr1G069780	0.37	1.01	−0.32
	At4g25090.1	HORVU5Hr1G078630	−1.91	2.29	−1.19
Peroxiredoxin (PRX)	At3g11630.1	HORVU2Hr1G073760	0.92	−0.50	0.94
	At3g26060.1	HORVU7Hr1G033500	0.85	0.00	1.09
	At3g52960.1	HORVU6Hr1G034620	0.73	−0.29	1.06
Superoxide dismutases (SOD)	At1g08830.1	HORVU2Hr1G021110	0.85	1.25	0.74
	At2g28190.1	HORVU7Hr1G060130	0.69	−0.13	1.15
	At5g51100.1	HORVU7Hr1G008390	0.11	−0.09	−1.39

List of main ROS genes differentially expressed in GP, Lum, and Tad plants. With the exception of NADPH oxidase (a ROS producer), all genes included in the table encode ROS-scavenging enzymes. fold change (Drought vs. control) is log_2_N. A fold change ≥ 1 are upregulated; between 0 < |fold change| < 1 are unchanged; fold change ≤ −1 are downregulated; *p*-value < 0.05.

## Data Availability

The data that support the findings of this study are available upon request from the corresponding author. The data are not publicly available due to privacy or ethical restrictions.

## Supplementary Materials

The following supporting information can be downloaded at: https://www.mdpi.com/article/10.3390/antiox12040790/s1, Figure S1: Quantitative real-time PCR validation of differentially expressed genes; Figure S2: Gene expression of the ROS-scavenging network in barley under drought; Figure S3: Transmission electron microscopy (TEM) analysis of the leaf cuticle membrane on GP, Lum, and Tad; Table S1: Primers used for qRT-PCR; Table S2: Light response curve and CO_2_ response curve parameters for GP, Lum, and Tad plants grown under glasshouse conditions; Table S3: Stomatal characteristics, determined by the photosynthesis measurements in the GP, Lum, and Tad; Table S4: Important differentially expressed genes in GP, Lum, and Tad under drought.
