# Supplementary material for: Stomatal Responses of Two Drought-Tolerant Barley Varieties with Different ROS Regulation Strategies under Drought Conditions
Source: Antioxidants (Basel). 2023 Mar 23;12(4):790. doi: 10.3390/antiox12040790 (PMC10135251; doi:10.3390/antiox12040790)
Supplement: Supplementary file 1 [file antioxidants-12-00790-s001.zip › antioxidants-2262618-supplementary.pdf]

Figure S1. Quantitative real-time PCR validation of differentially expressed genes.

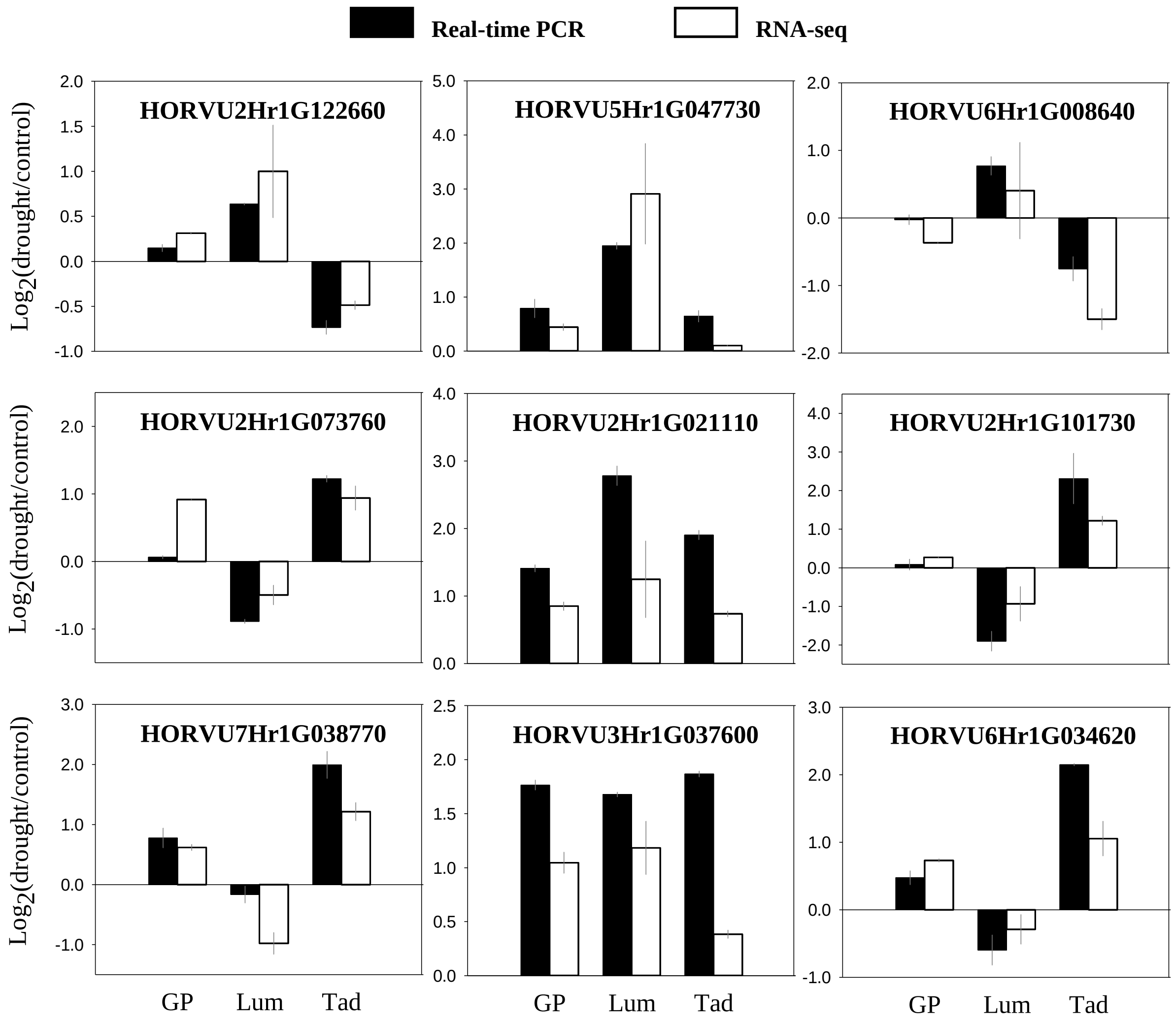

Expression patterns of nine DEGs are identified in Real-time PCR (black), RNA-Seq (white), in response to drought stress by qRT-PCR. GAPDH was used as internal control.

Figure S2. Gene expression of the ROS-scavenging network in barley under drought.

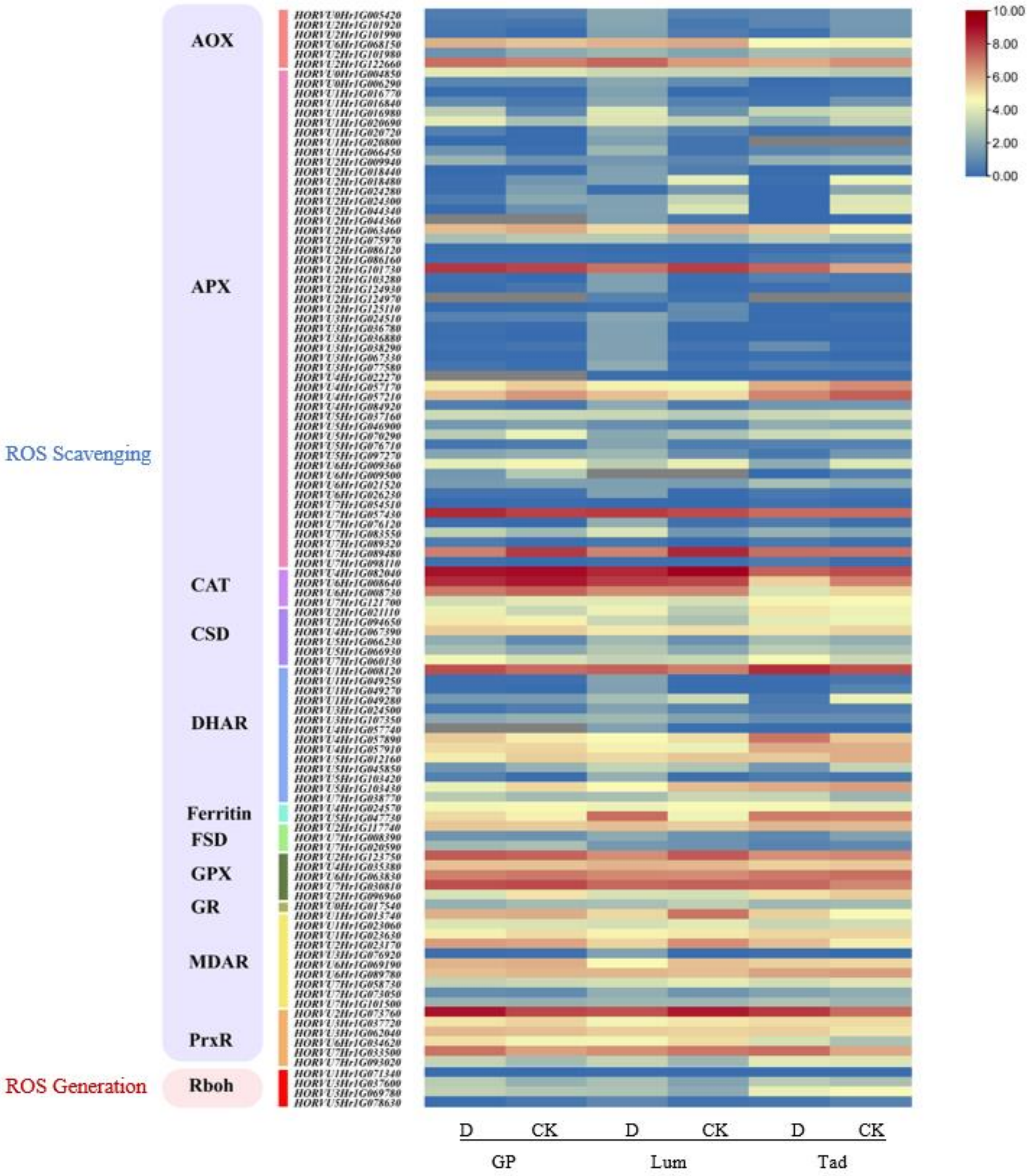

Main ROS genes and their corresponding enzymatic activities are listed. With the exception of NADPH oxidase (Rboh, a ROS producer), all genes included in the figure encode ROS-scavenging enzymes. The samples and treatments are displayed under each column.

Figure S3. Transmission electron microscopy (TEM) analysis of the leaf cuticle membrane on GP, Lum and Tad.

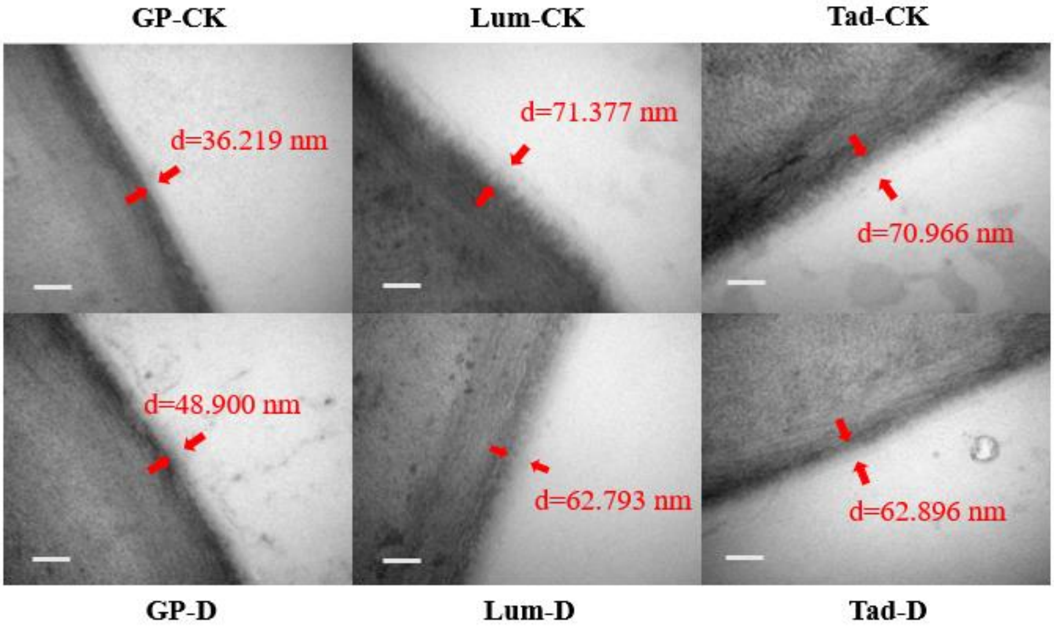

The leaf cuticle membrane was marked by red arrows, with thickness value (d). The samples and treatments are displayed on or under each column. Bar = 100 nm.

Table S1. Primers used for qRT-PCR.

Gene ID	Forward	Reverse
HORVU2Hr1G122660	AGCTCCAAATTCCAGGAGGC	CATCGTCTTGCAATGCTCCG
HORVU6Hr1G008640	CTCTACGACTCCATCGACGC	TCTGGCCATGTCTTGGTGTC
HORVU5Hr1G047730	CGTATCACTCCCTCTTCGCC	GGCATCACCTTTCTCGGGAT
HORVU2Hr1G073760	CATGGGTGCAGACAGAGAGG	GGTCTCATCCACACTACGGC
HORVU2Hr1G021110	GTCACCGGACTCAAGGAAGG	CATCTGTGCTGGAAGGGGAA
HORVU2Hr1G101730	CGAACCGGGGTGAGGAAATG	CGCCGATCAGATCCCTTCTG
HORVU7Hr1G038770	CCTACTCATGGCTGTCCTGC	CTGCGTGAAAGGACAATCGC
HORVU3Hr1G037600	TCGTTCCGCACTCATTGTCA	TGAGTCTTGACGCTGGTTCC
HORVU6Hr1G034620	GATGGACCTCTCCGACAAGC	AGGTTGAGCACCTTGACGAC
HvGAPDH	AAGCATGAAGATACAGGGAGTGTG	AAATTTATTCTCGGAAGAGGTTGTACA

Table S2. Light response curve and CO_2 response curve parameters for GP, Lum and Tad plants grown under glasshouse conditions.

	Control			Drought		
Parameter	GP	Lum	Tad	GP	Lum	Tad
Light compensation point (LCP, μmol m^{-2} s^{-1})	17.3523±2.4292	18.1567±0.6533	16.6174±0.9225	22.5166±6.8029	15.2695±1.1304	13.4743±0.9753
Light saturation point (LSP, μmol m^{-2} s^{-1})	3411.6095±828.0772a	4382.9040±537.7621a	3426.1838±328.6684a	993.6890±30.0411c	1734.272733±363.6590b	1256.95±185.1883bc
Rate of dark respiration (R_D, μmol m^{-2} s^{-1})	1.1058±0.1009a	1.1627±0.0349a	1.0305±0.0632a	1.0777±0.1767a	0.9319±0.0699ab	0.6841±0.0525b
Apparent quantum yield (AQY)	0.0674±0.0025a	0.0665±0.0008ab	0.0642±0.0008ab	0.0608±0.0077ab	0.0649±0.0013ab	0.0541±0.0016b
Light-saturated photosynthetic rate (A_{Lmax}, μmol m^{-2} s^{-1})	20.0962±1.2667b	24.4791±0.1823a	22.6972±0.4158ab	5.3035±1.1312d	12.2042±2.2115c	7.9249±1.0525d
CO_2 compensation point (μmol)	45.5298±0.1898cd	44.7224±0.3432d	45.6941±0.2196cd	63.9199±4.9419a	51.5657±0.7436bc	52.5431±0.8398b
CO_2-saturated photosynthetic rate (A_{Cmax}, μmol m^{-2} s^{-1})	20.0987±0.8756b	24.6869±1.0919a	19.4072±0.6156b	10.1791±1.5266d	17.1779±1.3232bc	14.2836±0.7962c
Maximum carboxylation rate of Rubisco (V_{cmax}, μmol m^{-2} s^{-1})	32.0940±2.5949c	38.0105±2.2577bc	39.4827±1.1446ab	32.9072±1.3504c	44.3749±1.0780a	36.3871±1.4370bc
Maximum electron transport rate (J_{max}, μmol m^{-2} s^{-1})	37.5742±2.9936c	43.765±2.7417bc	47.2829±0.7699b	42.244±3.5346bc	58.2533±0.4904a	43.7247±1.3139bc

The light-response curves and CO_2-response curves were fit for calculating LCP, LSP, R_D, AQY, A_{Lmax}, and Apparent CO_2 compensation point, A_{Cmax}, V_{cmax}, J_{max}. Data are presented as the means ± se (n=3-4), according to one-way ANOVA, followed with a Duncan's test for means comparison.

Table S3. Stomatal characteristics determined by the photosynthesis measurements in the GP, Lum and Tad.

	Control			Drought		
Parameter	GP	Lum	Tad	GP	Lum	Tad
g_s	0.2648±0.0017b	0.3338±0.0026a	0.3351±0.0033a	0.0770±0.0007d	0.1081±0.0010c	0.0805±0.0019d
$t_{1/2open}$ (min)	7.9264±0.3869a	7.3895±0.0902a	6.8846±0.7778a	10.3364±0.8684b	10.9624±0.4936b	10.8157±0.5217b
$t_{1/2close}$ (min)	7.2697±0.1116bc	13.7640±0.4333a	8.5046±0.4864b	4.7827±0.1867d	6.4565±0.5547c	2.9725±0.0302e

Dynamics of leaf-level stomatal conductance (g_s) were fit for calculating $t_{1/2open}$ and $t_{1/2close}$. Data are presented as the means ± se (n=3-4), according to one-way ANOVA, followed with a Duncan's test for means comparison.

Table S4. Important differentially expressed genes in GP, Lum and Tad under drought.

Gene ID	Fold change (Drought/control)			Annotation
	GP	Lum	Tad	
HORVU1Hr1G072210	2.40	-13.01	1.89	NAD-specific glutamate dehydrogenase
HORVU1Hr1G072780	0.29	2.70	0.02	Gamma-glutamyl phosphate reductase
HORVU1Hr1G088840	4.62	0.74	12.60	Phospholipase A1-II 7
HORVU2Hr1G034650	0.00	10.35	-9.78	zinc finger protein 7
HORVU2Hr1G057280	0.34	15.98	2.89	NAD-dependent epimerase/dehydratase
HORVU2Hr1G102840	0.25	1.74	0.27	Vacuolar cation/proton exchanger 1b
HORVU3Hr1G059170	1.44	5.76	1.22	Protein phosphatase 2C family protein
HORVU3Hr1G059220	-0.88	0.82	-2.52	WRKY family transcription factor
HORVU3Hr1G067360	0.31	12.35	-0.50	Protein phosphatase 2C family protein
HORVU4Hr1G022150	9.14	-11.64	3.55	Carbonic anhydrase
HORVU4Hr1G053130	-0.60	-2.58	-0.74	calmodulin 3
HORVU4Hr1G063980	0.53	1.94	0.42	Senescence/dehydration-associated protein-related
HORVU5Hr1G093700	1.85	6.08	0.87	lipoxygenase 1
HORVU6Hr1G009500	-2.00	0.00	-5.21	Peroxidase superfamily protein
HORVU6Hr1G055440	-5.41	-1.33	-7.68	RNA-binding protein 1
HORVU6Hr1G074440	0.28	2.18	0.35	annexin 7
HORVU7Hr1G076530	0.00	3.09	0.00	General transcription factor 2-related zinc finger protein
HORVU7Hr1G083550	0.88	3.24	0.47	Peroxidase superfamily protein
HORVU7Hr1G085010	0.82	2.24	0.37	Protein phosphatase 2C family protein
HORVU7Hr1G089320	1.99	11.69	1.67	Peroxidase superfamily protein

List of stomatal regulation, MAPK signaling, photosynthesis, and Ca^{2+}-transport related genes differentially expressed in GP, Lum and Tad plants. Fold change (Drought vs control) is log_2N, Fold change ⩾ 1 are up-regulated, between 0 < |Fold change| < 1 are unchanged and Fold change ⩽ -1 are down-regulated, Q-value < 0.001.
